# Removing the Mask of Average Treatment Effects in Chronic Lyme Disease Research Using Big Data and Subgroup Analysis

**DOI:** 10.3390/healthcare6040124

**Published:** 2018-10-12

**Authors:** Lorraine Johnson, Mira Shapiro, Jennifer Mankoff

**Affiliations:** 1 MyLymeData, Chico, CA 95927, USA; 2 Analytic Designers LLC., Bethesda, MD 20817, USA; mira.shapiro@analyticdesigners.com; 3 Paul G. Allen School of Computer Science and Engineering, University of Washington, Seattle, WA 98195, USA; jmankoff@uw.edu

**Keywords:** Lyme disease, real-world evidence, patient-centered research, patient-reported outcomes, treatment heterogeneity, average treatment effect, global rating of change scale, individualized care, patient registries, big data

## Abstract

Lyme disease is caused by the bacteria borrelia burgdorferi and is spread primarily through the bite of a tick. There is considerable uncertainty in the medical community regarding the best approach to treating patients with Lyme disease who do not respond fully to short-term antibiotic therapy. These patients have persistent Lyme disease symptoms resulting from lack of treatment, under-treatment, or lack of response to their antibiotic treatment protocol. In the past, treatment trials have used small restrictive samples and relied on average treatment effects as their measure of success and produced conflicting results. To provide individualized care, clinicians need information that reflects their patient population. Today, we have the ability to analyze large data bases, including patient registries, that reflect the broader range of patients more typically seen in clinical practice. This allows us to examine treatment variation within the sample and identify groups of patients that are most responsive to treatment. Using patient-reported outcome data from the MyLymeData online patient registry, we show that sub-group analysis techniques can unmask valuable information that is hidden if averages alone are used. In our analysis, this approach revealed treatment effectiveness for up to a third of patients with Lyme disease. This study is important because it can help open the door to more individualized patient care using patient-centered outcomes and real-world evidence.

## 1. Introduction

Lyme disease, caused by the spirochete *Borrelia burgdorferi* and transmitted via tick bite, is the most common vector-borne disease in the United States. The Centers for Disease Control and Prevention (CDC) estimates that 300,000 new cases of Lyme disease occur annually [1]. Although most patients who are diagnosed and treated early are restored to health, treatment failures ranging from 10% to 35% have been reported, and many patients are not diagnosed until later in the disease when treatment success is much harder to achieve [2,3,4,5,6,7,8,9]. Compared to other diseases, very little research has been conducted on how best to treat patients who do not respond to short-term treatment approaches [10].

Different nomenclature has been used to describe this group of patients. In this paper, we use the term chronic Lyme disease, which is the name most commonly used by patients and their treating clinicians [11,12,13]. The term “Post Treatment Lyme Disease Syndrome” (PTLDS), which was first proposed in 2006, incorporates the restrictive CDC surveillance criteria. However, as the CDC notes, the goals of surveillance and clinical diagnosis are very different [14,15]. While restrictive case definitions may be useful for surveillance or for specific research purposes, they exclude a large portion of the clinical population of patients whose symptoms persist despite their having received some antibiotic therapy. Hence, surveillance-based definitions should not be used by healthcare providers for making a clinical diagnosis, and the generalizability of definitions based on these criteria to the clinical population is limited [15].

Just three research studies have been funded by the National Institutes of Health (NIH) on the retreatment of chronic Lyme disease, and the last retreatment trial was funded over 15 years ago [16,17,18]. The patients enrolled in these three randomized controlled trials were from highly selective and small samples. The trials produced conflicting results and relied on average treatment effects as their measure of success. This has created considerable uncertainty in the treatment of patients with chronic Lyme disease.

While randomized controlled trials and average treatment effects contribute to our knowledge base, they can also mask important information that can provide insights into the range of treatment responses existing within a patient group. For example, although it has long been contended that tick-borne coinfections are rare in patients with Lyme disease [19], coinfections are common in patients with persistent Lyme disease symptoms [5]. One reason patients may develop chronic disease is *because* they are coinfected with other tick-borne pathogens. A better understanding of the difference between acute and chronic Lyme disease characteristics could identify patients with a heightened risk of developing chronic disease, and awareness of these risk factors might prompt more aggressive treatment in those patients—towards a goal of preventing the development of chronic Lyme disease.

Using a big data sample of almost 4000 people diagnosed with Lyme disease from the MyLymeData patient registry developed by LymeDisease.org, we looked at responses to a widely used global rating of change (GROC) scale to assess the degree to which patients reported that their condition improved, worsened, or remained unchanged following antibiotic treatment. The survey questions used were both patient-centered and reflective of the types of questions posed by clinicians in practice. To study treatment response, we created subgroups of patient-reported changes in the condition of their health. Our findings indicated that more than a third of people with Lyme disease in this group of patients were “high responders” to antibiotic treatment, suggesting the potential value of subgroup analysis as a standard component of future study designs.

Our big data sample included the experience of a much broader group of patients than were included in previous Lyme disease clinical trials [11,12,13]. In addition, as the central participants in the healthcare process, patients can draw upon many sources of personal health data that are traditionally locked in separate electronic health records, insurance records, clinician notes, or research silos. Thus, data collected from patients, while self-reported (and thus to some degree subjective), is also more complete than many of the other sources. This approach complements randomized controlled trials (RCTs), which can provide the high internal validity essential to demonstrate cause and effect but may do so at the expense of generalizability to clinical care of individual patients [20,21]. This is because as a research design, randomized controlled trials may employ highly restrictive entry criteria to eliminate factors that could be confounding (such as other potential causal agents for change) and rely on average treatment effects to demonstrate treatment effectiveness. This inherently limits the generalizability of these types of trials to the clinical population, with some trials excluding up to 95% of people who might use the intervention [20]. In contrast, our approach not only encourages broader study participation, which increases the applicability of the results to the patient population seen clinically, but also produces larger sample sizes that enable the type of robust subgroup analysis necessary to identify treatment response variation within the group.

Our subgroup analysis is important because it can help open the door to a more personalized approach to patient care. In healthcare, the primary goal is to improve outcomes that are important to individual patients. Clinicians are less interested in what works for the average patient than what is likely to work for their particular patient given the patient history, severity of presentation, comorbidities, treatment responsiveness, and ability to tolerate treatment side effects [20]. Clinicians require information about real-world outcomes that reflect the heterogeneity of treatment response typically seen in clinical care. Subgroup analysis of big data samples is therefore important as it may help facilitate a more personalized approach to treatment of Lyme disease patients [20]. Over the past ten years, the technological tools required to pool large amounts of data have become faster, cheaper, and more readily available.

## 2. Materials and Methods

### 2.1. Data Sources

This study analyzed the responses of those who completed Phase 1 of the MyLymeData project. Survey items used in Phase 1 were drawn from the Agency for Healthcare Research and Quality’s (AHRQ, Rockville, MD, USA) recommended data elements for registries, prior surveys, and peer-reviewed published literature for Lyme disease and other conditions [4,5,21,22]. (Appendix A generally describes registry items included in MyLymeData). We also relied heavily on survey questions derived from standard government question banks, such as the CDC Behavioral Risk Factor Surveillance System, National Health Interview Survey, National Ambulatory Medical Care Survey, and National Center for Health Statistics as well as the AHRQ Medical Expenditure Panel Survey [23,24,25,26,27,28,29]. Specific items, including the GROC scale to assess whether a patient’s health condition has improved or deteriorated over time, were based on published studies using GROC as discussed further below [30,31,32]. The beta version of the MyLymeData survey was pilot-tested and adjusted as recommended by AHRQ.

To promote participation in the survey, various recruitment strategies were utilized, including blogs and social media as well as professional conference presentations about the registry. Participation in the registry is voluntary, and all respondent identities remain strictly confidential. The registry and survey was approved by the Advarra Institutional Review Board at the time it was launched. Analysis of the survey data (see Table S1) was exempted from review by the Washington University Institutional Review Board (IRB) because none of the data used for this study contained personally identifiable information. SPSS and SAS^®^ JMP Pro^®^ 13.1.0 were used to conduct this analysis. 

### 2.2. Study Participants

Unlike randomized trials, which have highly restrictive entry criteria, the enrollment criteria for patient registries is generally expansive and includes US patients who self-report being diagnosed with a particular condition [33]. Within the patient registry sample, further patient selection can be done by stratifying patients at risk of disease progression (e.g., stage of illness at diagnosis or presence of coinfections) or by diagnostic validation criteria [5]. Commonly used Lyme disease diagnostic validation criteria include exposure, recollection of a tick bite, presence of an erythema migrans (EM) rash, signs or symptoms consistent with Lyme disease, positive laboratory tests, and clinician diagnosis. Because the goal of this study was to establish the usefulness of GROC scales as a proof of concept to describe treatment heterogeneity and identify treatment responders, this study focused on patients who had been diagnosed with Lyme disease by a healthcare provider, regardless of diagnostic method. Future studies could confirm these findings with a more restrictive patient sample. For example, a previous study by the authors examined survey respondents who either had an EM rash or two-tiered CDC positive testing [5]. Appendix B lists additional criteria used in a clinical research definition of chronic Lyme disease that might be used to create a more strictly characterized research sample [34].

A total of 5872 patients had enrolled in Phase 1 of the registry study, which had a rolling enrollment period. Patients who had either not completed their registration or had not completed all their Phase 1 surveys were rolled forward into the Phase 2 survey when it launched. A total of 4719 had completed registration and all surveys for Phase 1.

In addition to completing the Phase 1 surveys, participants were required to answer “yes” to questions in the baseline survey confirming that they were residents of the US and that they had been diagnosed with Lyme disease by a healthcare provider.

At the end of the baseline survey, participants classified their current health status as either well or unwell and were given an additional survey based on their response to that question. After completing the baseline survey, participants were required to complete either the unwell or well survey.

Hence, the preliminary sample of 4719 participants who had completed all required surveys was subsequently reduced to 3903 after eliminating duplicate participants (through their unique registration identification number) as well as those who did not designate a US state of residency or report diagnosis of Lyme disease by a healthcare provider. Of the final sample, 3556 completed the unwell survey and 447 completed the well survey. 

**Figure 1:**
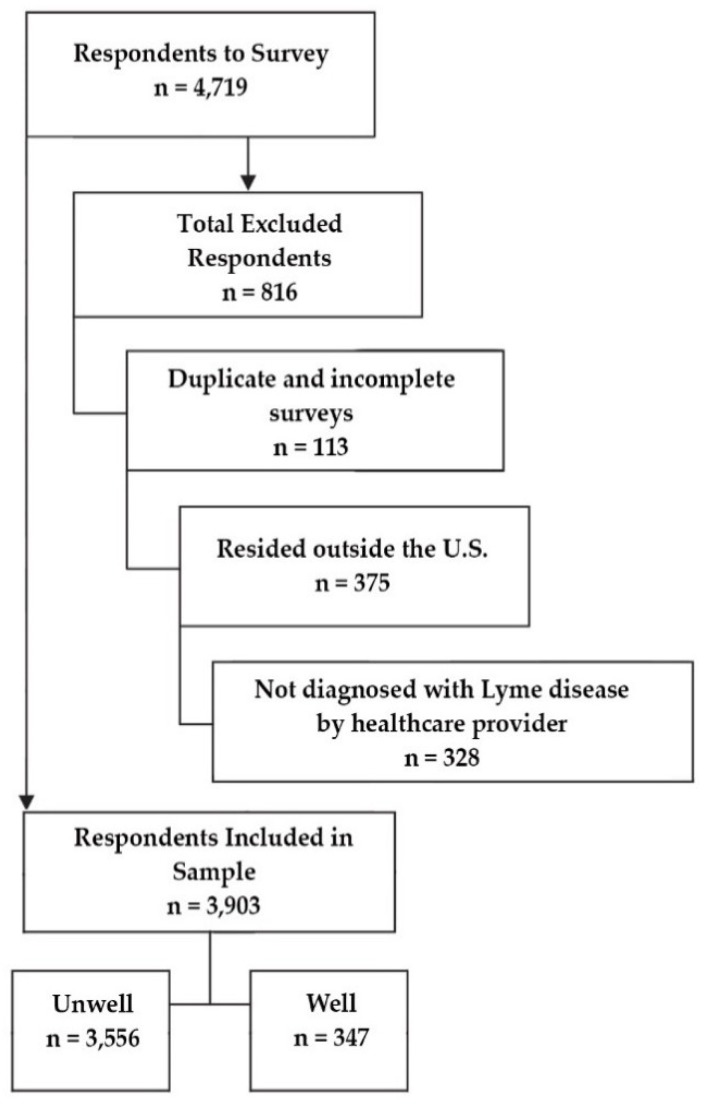
Preliminary sample, exclusions, and final sample size determination.

Figure 1 shows the original sample of 4719 respondents and the exclusion criteria that led to a final sample of 3903 subjects. The 3903 participants used in this analysis included US residents clinically diagnosed with Lyme disease who had completed the Phase 1 survey and the unwell or well survey assigned to them.

Table 1 shows the demographic characteristics of the resulting group of patients. The sample here was predominantly female (83%), and the average age was 49. This sample also skewed more towards higher education and income levels than the general population. Note that while all participants completed the baseline (which covered topics such as diagnosis, tick exposure, and early symptoms), only those patients who reported their status as unwell were served the GROC survey questions and were asked to identify their current three worst symptoms. Of these 3556 respondents, 20 failed to complete the GROC survey questions and were excluded in that analysis.

Table 2 below shows the current stage of illness of those in the sample as well as some of their diagnostic characteristics. These diagnostic characteristics and stage of illness criteria might be used to further refine a sample to conform to a recently proposed research definition of chronic Lyme disease [34]. (See Appendix B).

### 2.3. Global Rating of Change Scale

An important focus of our work was to take a patient-centered approach. Outcome measures that are patient-centered reflect the patient’s perspective on response to treatment. In Lyme disease, these include restoration of health, prevention of health deterioration, ability to engage in work and other activities, and improvement in quality of life [28]. GROC scales allow the patients themselves to determine what factors are important in assessing their condition [31]. Because they are useful in observational trials, clinical practice, and randomized controlled trials, they provide a bridge of interoperability among these three critical arenas. GROC scales also make it possible to employ a highly granular approach that can help differentiate treatment responses within a group.

GROC scales are widely used in both research and clinical practice [35,36]. The face validity of GROC scales is high as they are intuitive and easy to understand for patients, clinicians, and researchers alike [37]. They can also be used to collect treatment response data from a variety of sources, including patient registries, clinicians, and researchers with high levels of concordance and allow an anchoring point of time for comparison that can lend itself to different treatment durations [20]. Scores obtained using GROC scales have been found to correlate with pain, disability, and quality of life measures [38].

GROC scales ask patients to identify whether their symptoms are better, worse, or unchanged from a previous point in time and utilize a Likert scale ranging from 7 to 17 points to assess the granularity of treatment response [30,35]. Highly granular outcome measures permit treatment response to be categorized more accurately [38]. Outcome measures also provide objective measurement of treatment response for clinical trials and may help clinicians monitor treatment effectiveness and allow researchers and clinicians to predict which patients will benefit most from a particular intervention [38].

We used a common 17-point Likert scale to explore the potential use of this approach to unmask subgroup variation of treatment response compared to the average treatment effect [36]. To make the scale more patient-centered, we followed the lead of an earlier study that asked the question in a two-step process [32]. First, patients were given the choice of responding “better”, “worse”, or “unchanged” to the statement “In general overall, I would say that with antibiotic therapy, my Lyme symptoms are ___.” Those who responded “better” or “worse” were asked a follow-up question regarding the magnitude of change perceived by the patient on an 8-point scale. A 17-point scale presented as one question would likely be difficult for a patient to answer, while the two-step question approach used here reduced that cognitive load.

The end product of this two-step or branched question sequence was a 17-point Likert scale, ranging from −8 to +8, with the unchanged midpoint pegged at 0. For example, compared with no change, those responding “better” would select a magnitude ranging from (a) “almost the same”, (b) “hardly better at all”, (c) “a little better”, (d) “somewhat better”, (e) “moderately better”, (f) “a good deal better”, (g) “a great deal better”, and (h) “a very great deal better”. Those responding “worse” would choose among similar responses in the negative (e.g., “hardly worse at all” to “a very great deal worse”) [32].

In our responses, very few patients (2%) selected “almost the same”, and the distribution was evenly split between those who reported being better or worse (1% each). In conformity with other studies, we elected to include the response “almost the same” in the unchanged category, thereby reducing the final scale to a 15-point scale ranked +1 to +7, ranging from “hardly better to all” to “a very great deal better” on the positive end of the scale and −1 to −7 on the negative end of the scale for comparable categories [30].

We additionally grouped these responses as Better, Unchanged, and Worse and further characterized those responding as High Responders (score between 4 and 7), Low Responders (score between 1 and 3), and Nonresponders (score between −7 and 0). We chose a cut-score of +4 or greater to define High Responders based on the assumption that most patients would regard a treatment response of “moderately better” to “a very great deal better” as an important improvement.

Prior work suggests that the appropriate cut-off to use is disease-specific, with cut-offs ranging from +1 to >+3 or greater based on the amount of change regarded as important to patients and clinicians [30]. Some studies ask patients to specifically identify what degree of improvement they regard as the minimally important amount of change or minimally important clinical difference [32]. Indeed, the scale we used was derived from a study by Jaeschke and Guyatt to ascertain minimal clinically important difference (MCID) [36]. This study did not seek to establish the MCID for Lyme disease, although future studies may do so.

## 3. Results

### 3.1. General Observations

Misdiagnosis and delayed diagnosis were recurring themes in our sample, which consisted predominately of patients with chronic Lyme disease (61%) who reported having remained ill for six or more months after 10–21 days of antibiotic treatment. More than half (51%) reported that it took them more than three years to be diagnosed and roughly the same proportion (54%) saw five or more clinicians before diagnosis. These diagnostic delays occurred despite the fact that 45% of participants reported early symptoms of Lyme disease within days to weeks of exposure. Causes of identified diagnostic delays included false negative lab tests (37%) or positive test results that were dismissed as “false-positives” (13%). While delayed diagnosis appears to be common in those with chronic Lyme disease, the extent of the problem for the full disease spectrum is unknown. However, some researchers suggest that delayed diagnosis may occur in as many as 40% of cases [3].

The majority of patients (72%) reported being misdiagnosed with another condition prior to their Lyme diagnosis. Of those misdiagnosed, the most common misdiagnosis was psychiatric disorder (52%), followed by fibromyalgia (43%), followed by chronic fatigue (42%). Although misdiagnosis with a psychiatric illness was common (52%), only (18%) reported psychiatric symptoms as being among their three worst symptoms.

The most frequently reported three worst symptoms included neurologic-associated symptoms (84%) and fatigue (62%), followed by musculoskeletal-associated symptoms (57%). Neurologic symptoms included cognitive impairment, sleep impairment, memory loss, psychiatric manifestations, headaches, neuropathy, and twitching, with cognitive impairment (30%) and neuropathy (29%) most often reported among those with neurologic symptoms. Musculoskeletal symptoms included muscle aches and joint pain.

Symptomatic relief using other prescription medications was associated with some of the commonly reported symptoms. For example, sleep impairment was widely reported as among the top three worst symptoms (20%), with sleep medications taken at higher rates (34%) than the general age-adjusted population (9%) [39]. Thyroid medication, which is sometimes associated with managing fatigue, was also much higher (33%) than the general age-adjusted population (8%) [39]. Similarly, pain-associated symptoms (joint pain, muscle aches, or neuropathy) were common (71%), and prescription pain medication usage (26%) was higher than the age-adjusted rate in the US general population (16%) [39].

Most patients did not recall or did not know if they had a tick bite (59%) but 29% of those who did, reported that the tick was attached for less than 24 h. Very few (2%) had the tick tested for pathogens. The majority (78%) reported that their Lyme disease diagnosis was supported by positive lab tests, and 45% of these reported that their tests were positive by either CDC two-tiered or western blot interpreted by CDC banding criteria.

Consistent with a prior study published in 2014, coinfections appeared to be common (60%; 45% with positive lab test confirmation), rather than the exception [5]. Although the rate of laboratory-confirmed coinfections in this sample was slightly lower than reported in the 2014 study (45% vs. 53%), the difference likely reflects differences in the inclusion criteria between the two samples.

The most commonly reported coinfections were Babesia (23% with supporting lab tests, 21% without) and Bartonella (19% with supporting lab tests, 23% without). Reported rates of Ehrlichia or Anaplasma were substantially lower (11% with supporting labs, 5% without). (Note that the rate of Bartonella in the general population is reported to be 3–6% [40] but has been reported to be as high as 28% in higher-risk populations such as veterinarians [41]. The 19% rate of positive Bartonella serology in our sample suggests that patients with chronic Lyme disease may be at higher risk of acquiring this coinfection.) These coinfection rates should be viewed in the context that many physicians do not test for coinfections and those who do, may only test for select coinfections.

Consistent with other studies, a majority (65%) reported their health status as fair or poor [5,17]. Moreover, 32% reported their work status as disabled (whether or not receiving disability payments). Finally, the great majority reported being treated by internists, family practitioners, or physicians who specialize in the diagnosis and treatment of tick-borne diseases. Few (6%) reported being treated by infectious disease physicians.

### 3.2. Global Rating of Change Scale

We compared the responses to the GROC scale with the average treatment effect to determine if this highly granular Likert scale was able to detect subgroups of patients who responded better or worse than the mean.

As shown in Table 3 below, 17.26% of the responses translated to a Likert scale value of 1 (“hardly better at all”), 2 (“a little better”), or 3 (“somewhat better”); 34.64% of patient responses were 4 or higher (“moderately better”, “a good deal better”, “a great deal better”, “a very great deal better”), and the remaining 48.11% of responses were 0 or lower (“unchanged” or from “hardly worse at all” to “a very great deal worse”).

**Figure 2:**
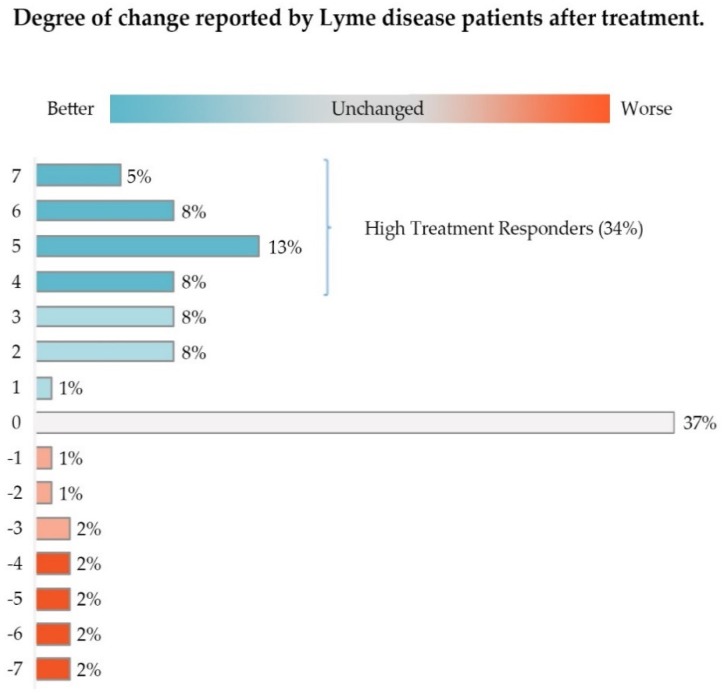
The majority of participants (51%) reported some improvement in their condition after treatment with antibiotics, with High Treatment Responders constituting 34% of participants. Approximately 37% reported their condition as unchanged. Only 12% reported their condition as worse. Slight deviation in the percentages in the figure from the text reflect rounding errors.

Approximately 52% of this group of patients (Low and High Responders) reported some improvement in their condition. As shown in Figure 2, High Responders (Likert score of 4–7) made up 34.64% of study participants, while Low Responders constituted 17.26%. About 36% of these patients reported their healthcare status as unchanged; less than 12% reported that their condition was worse.

**Figure 3:**
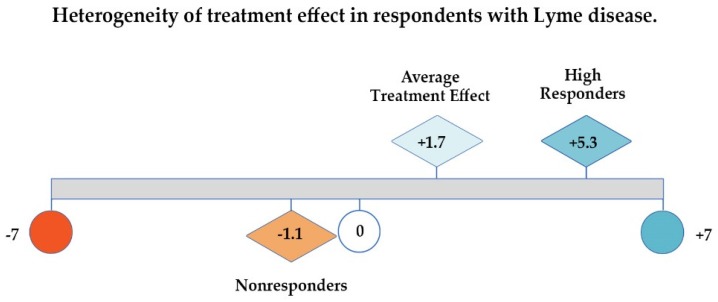
Average treatment effect, high responders, and nonresponders on global rating of change (GROC) scale shows heterogeneous treatment response among participants that average treatment effect masks.

As Figure 3 below illustrates, if patient responses are evaluated by looking at the GROC average treatment effect or mean Likert score alone (1.7), one might conclude that there has been very little improvement in the health of this sample of unwell patients; using only the median (2.0) would lead to a similar conclusion. However, examining the distribution of the values for the unwell patients unmasks significant improvement (5.3 points out of 7 on a 7-point scale) among the 35% who were High Responders. Very few patients reported worsening (12%), and the deterioration among total Nonresponders as a category (which includes those who were unchanged) averaged −1.1 points out of 7.

Rather than merely calculating measures of central tendency as treatment effectiveness outcomes, using finer granularity of patient response enhances the ability to detect treatment response. Examining characteristics of this subgroup may lead to greater insights about risk identification of different groups of patients and help predict which patients are most likely to respond to treatment. 

## 4. Discussion

### 4.1. The Value of Patient-Generated Health Data

LymeDisease.org, a grassroots nonprofit organization that supports the interests of Lyme disease patients, has conducted and published peer-reviewed big data patient surveys for over 10 years [4,5]. In November 2015, it launched a patient-powered registry, MyLymeData, that enables patients to pool their healthcare data as a community research resource [42]. Patient-powered registries are similar to researcher-generated patient registries, but the registry is managed and controlled by patients who set the research agenda for the data [42,43].

The purpose of MyLymeData is four-fold: to conduct observational research, to assist researchers in conducting and recruiting for traditional and innovative clinical trials, to improve the quality of care through standard of care studies, and to create healthcare policy change. Since its launch, over 11,000 patients have enrolled, and the National Science Foundation awarded a grant to a team of researchers to explore data analytic techniques using registry data.

Patient-powered registries rely primarily on patient-generated data, which is defined by the Patient Outcomes Research Institute as:

“Health-related data—including health history, symptoms, biometric data, treatment history, lifestyle choices, and other information—created, recorded, gathered, or inferred by or from patients or their designees (i.e., care partners or those who assist them) to help address a health concern.”

[44]

Patient-generated data is increasingly recognized as a valuable source of real-world evidence by government projects, including PCORnet (launched by the Patient Centered Outcomes Research Institute), the NIH Collaboratory, the CDC National Amyotrophic Lateral Sclerosis (ALS) Registry as well as professional, commercial, and patient organizations such as the American Society of Clinical Oncology, PatientsLikeMe, CancerBase, and the Duchenne Registry [20,44,45,46,47,48,49].

Big data analytics are expected to play a critical role in the emergence of personalized medicine and individualized care. The NIH notes that patient registries can help

- improve recruitment,
- identify patient research cohorts,
- conduct natural history studies,
- integrate patient-reported and clinical data from multiple sources into single registry,
- stimulate new research on the causes, treatments, and outcomes of diseases,
- accelerate research, knowledge discovery, and scientific insights from patients with under-researched diseases, and
- enhance creative data mining within and across diseases [50].

Patients hold an enormous amount of inexpensive, underutilized data. Unlike traditional trials, patient registries permit researchers to

- enroll diverse patient populations,
- evaluate care as it is actually provided in real-world practice,
- assess complex treatment patterns and treatment combinations, and
- offer the ability to evaluate patient outcomes when clinical trials are not practical or are difficult to conduct (for example, when long-term outcomes are important) [22].

To harness the full benefits of personalized healthcare will require more efficient research practices and big data analytics to discover deep knowledge about patient similarities, personalized disease risk profiles for individual patients, and treatment response heterogeneity [51].

### 4.2. The Need to Accelerate Research in Treatment of Lyme Disease

**Figure 4:**
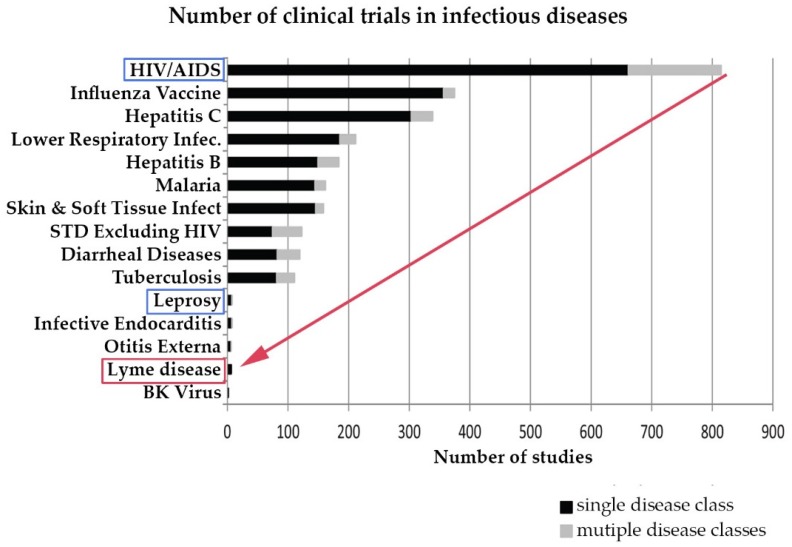
Research in Lyme disease is sparse compared to other infectious diseases. (Derived from Goswami 2013 [10]).

Research on chronic Lyme disease needs to move forward at a much more rapid pace than it has historically. Although Lyme disease is a common disease, until the CDC revised its estimated annual incidence to 300,000 cases in 2013, this was not commonly recognized, and very few clinical studies of Lyme disease have been conducted compared to other infectious diseases. The CDC estimates that the annual incidence of Lyme disease is 300,000 and has grown over 300% since the late 1990s. However, although the incidence of Lyme disease is nearly 8 times higher than the number of people diagnosed with HIV/AIDS each year in the US (38,500), the number of clinical studies for Lyme disease trails behind leprosy, which has an incidence of less than 200 cases a year [52,53,54,55]. This is illustrated in Figure 4 below derived from a study by Goswami on clinical trials for infectious diseases listed on ClinicalTrials.gov [10].

The few clinical trials that have received NIH funding on Lyme disease have been hampered by highly restrictive selection criteria for study participation, leading to very small samples (ranging from 37 to 129) and long recruitment times (2.5 to 4 years). Table 4 delineates these factors and compares the NIH trials with a big data study of over 3000 patients recruited in six months. The big data study detailed in the table included chronic Lyme patients (a) who were clinician diagnosed, (b) had either an EM rash or positive serology, and (c) who remained ill for six or more months following treatment with antibiotics.

The largest randomized controlled NIH trial screened out 93% of the 1966 patients who attempted to enroll [17]. In trials with small sample sizes, it is difficult to detect small or moderate treatment effects that most patients would regard as meaningful [56,57]. This was highlighted in an important critique of the Klempner trial [58]. Ultimately, these trials are not generalizable to most patients seen clinically and are too small for subgroup analysis.

### 4.3. Samples and Outcomes in Lyme Disease Studies Reflect a Heterogeneous Patient Population

There are a wide range of factors that may contribute to heterogeneity among patients with Lyme disease in either severity of illness or responsiveness to treatment. Patients with chronic Lyme disease have a variety of symptoms, incidence of coinfections, and delays in diagnosis. In addition, strain variation and epigenetics likely play a role in disease presentation. Heterogeneity of treatment response may arise from individualized risk of disease progression, responsiveness to treatment, vulnerability to adverse side effects from treatment, the person’s unique assessment of trade-offs involved in different health states, and the treatment-associated risks and benefits (often referred to as utilities) [59].

To further compound the problem, we do not yet fully understand the distribution of the population of Lyme disease patients because the full spectrum of the disease is not yet well understood. In Lyme disease, many studies focus on acute, well-characterized patients who evidence objective manifestation that the CDC uses for surveillance purposes [19] or other similarly restrictive definitions of Lyme disease [15,60]. This may be useful for randomized controlled trials and surveillance purposes where being highly selective achieves an associated goal. However, this approach does little to inform us about the characteristics of the disease that do not meet that narrow definition, for example, a patient without a rash or Bell’s palsy or who does not test positive on two-tiered serology. Thus, other more inclusive research definitions of Lyme disease that reflect those used in clinical care might prove useful in real-world clinical trials [34]. (See Appendix B).

Prior work by the authors compared restrictive diagnostic criteria on a sample of 3000 patients with either an EM rash or serological evidence for Lyme disease [5]. This work found that patients who met the CDC vs. less restrictive laboratory diagnostic criteria were similar demographically and on other outcomes measures used in that study. However, patients who did not meet the more restrictive CDC definition were more likely to have their diagnosis delayed. This is problematic because a preliminary analysis of patient outcomes from the same MyLymeData dataset used in this paper found evidence suggesting that patients diagnosed early were far more likely to be well than those with a delayed diagnosis [61].

Hence, patients who experience delayed diagnosis may be more likely to develop chronic Lyme disease [62,63]. For example, females may be at higher risk of contracting chronic Lyme disease due to diagnostic delays if laboratory diagnostic testing is more effective in males, as has been suggested [64,65]. They may also be at higher risk of developing chronic Lyme disease generally as indicated in a large, insurance-based big data study [66]. These factors may explain why our sample was predominantly female.

However, a less restrictive definition of Lyme disease alone is not sufficient to inform treatment decisions because treatment response may vary in different subgroups. Identifying subgroup variation requires not only larger sample sizes but also the recognition that assessing treatment response using average treatment effects will fail to capture treatment response heterogeneity and may overlook minimum clinically important differences for the disease [58].

**Figure 5:**
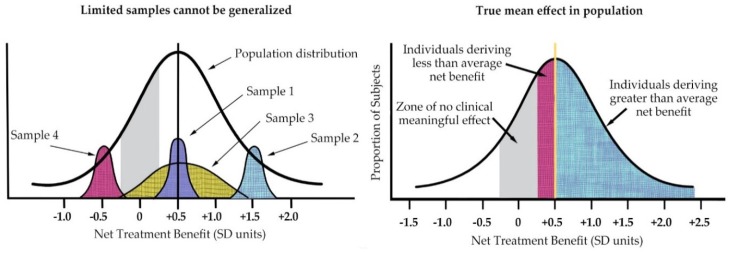
(**left**) Treatment effects of nonrepresentative samples cannot be generalized to the full spectrum of disease; (**right**) In addition, within any given sample, individual patient treatment response varies from mean. (Derived from Kravitz 2004).

As Kravitz illustrated (see Figure 5 below), the individual patient seen in clinical practice is not average but unique in terms of treatment response [22]. He also highlighted the fact that averages can only reflect the sample population of a particular study and will change with different patient populations to reflect the study entry restrictions.

Misapplying averages to a heterogeneous group of individual patients, such as those in our study, can create harm by either providing treatment that is unlikely to benefit a patient or by denying a patient treatments that would be beneficial to them [59]. Unfortunately, treatment guidelines often base their recommendations on average treatment effects. Some commentators contend that average treatment effects should not be used to constrain individualized care unless the studies used by guideline committees are sufficiently powered to detect and rule out treatment response heterogeneity [37]. However, medical decisions are made for individuals, and assessment of the heterogeneity of treatment effects is critical as medicine seeks to become more personalized and patient-centered [67].

Our analysis compared the average treatment response with subgroups of patients who were categorized as High Responders, Low Responders, and Nonresponders. Using the GROC scale to assess treatment response, rather than studying average treatment effectiveness, we demonstrated the value of subgroup analysis to unmask heterogeneous treatment response within a sample. Although average treatment response for the sample showed negligible improvement, the GROC scale permitted a more granular analysis that differentiated treatment responders in the sample. This approach indicated that a majority of those participating in the study (approximately 52%) improved with treatment, and a significant subgroup (approximately 35%) were High Responders, which is in line with the effectiveness of therapies in other diseases [68]. This is important because a better understanding of the 1 out of 3 patients who respond well to treatment can potentially guide the development of treatment mechanisms and allow a more personalized approach to treatment.

This study demonstrates the power of using patient data to better understand the course of disease and the success of treatment. The highly granular classification value of the GROC scale when used with a large data set is demonstrated as this tool was able to successfully delineate Lyme disease patients into subgroups according to their response to treatment. To our knowledge, this is the first published study using patient-generated data from a patient registry to assess GROC in a big data study. We suggest more research is needed to assess the applicability of using patient-reported data and clinical data on patients with chronic Lyme disease to discover what illness, patient, and treatment characteristics may lead to improvements in health status. This research is critically important to inform the development of medical guidelines for chronic Lyme disease that are more patient-centered in their approach.

An understanding of the characteristics of the underlying sample may also prove useful in developing strategies to prevent patients from progressing to the persistent form of the disease through earlier or more targeted intervention. For example, diagnosing patients earlier or using more aggressive forms of intervention for those at higher risk, such as patients who had delayed diagnosis or coinfections, might improve treatment outcomes.

### 4.4. Strengths and Limitations

All data sources have their strengths and limitations depending on their source and characteristics. Hence, great care needs to be taken for comparison purposes and for any meta-analyses. For example, convenience samples drawn from clinicians may have inherent sample bias reflecting the geographic location of the office as well as the treating style of the physician (and whether patients select to be treated by the physician). Insurance databases are broad but are limited to the data they collect and only include those whose treatment is covered by the insurance plan. (Many Lyme patients report that their physicians do not accept insurance.) Electronic health records may be limited by their intended utilitarian focus (billing claims). Appendix C lists various data sources and their limitations.

Applying average treatment effects from small, highly selective randomized trials to the clinical population is problematic because of the lack of generalizability of these trials. Many clinical trials conducted in the US report on average treatment results, with some concluding that there is no treatment effect [59]. In essence, these studies are reporting on results for the “average” patient despite the variation in patient characteristics and outcomes seen in clinical practice [69].

Because patient registries reflect real-world behavior and practices and employ fewer inclusion and exclusion criteria, they are more inclusive than randomized controlled trials and more generalizable to patients seen in clinical practice [70]. Patient registries are also not tied to a geographic locus and may hence reflect greater geographic diversity than traditional research trials previously conducted.

However, registries also pose unique challenges because patients are recruited directly in situations where the underlying sampling frame is unknown and registry samples are often self-selected (as is the case with MyLymeData) [70]. These participants have access to the internet and are not a randomly drawn sample. Those who elect to participate may have been sick longer and may have been more severely ill, which could lead them to seek online support and resources for their illness [5].

The patient registry results presented here are based on self-reported information without independent diagnostic confirmation. However, self-reported information is reported to improve accuracy of patient data and has been found to have acceptable levels of reliability when compared to medical chart information [70,71,72,73,74].

MyLymeData has a relatively low rate of participation from those with early disease (6%). This suggests that patients without chronic disease may have less motivation to join and see less need to pool data assets as a community. For example, patients with a short-lived acute illness like the common cold have no need for community support; they simply get on with their lives. Accordingly, we believe that the results reported here capture a segment rather than the spectrum of those with Lyme disease.

Finally, although this study included data from close to 4000 participants in MyLymeData and has significant implications for research design and health policy, observational samples are not suitable for determining cause and effect.

## 5. Conclusions

The focus of this study is to demonstrate the usefulness of the GROC scale to unmask heterogeneous treatment effects in Lyme disease. In Lyme disease, researchers have emphasized average treatment effects when assessing the effectiveness of treatment interventions [16,17,18]. However, averages may mask heterogeneity of treatment response by failing to distinguish between patients who respond better or worse than average [59]. The purpose of examining treatment response heterogeneity is to improve individualized care and health outcomes that are most meaningful to patients and clinicians [37,70].

The analysis discussed in this paper, using a Likert scale to provide a more granular view of treatment response, demonstrates the benefit of going beyond examination of average treatment effects. With the increased availability of big data—both patient-generated and clinician-generated—a highly granular approach is becoming more feasible.

Determining the best clinical treatment approach for an individual is fundamentally different from determining the average treatment effect in a randomized clinical trial, although the two are often conflated [37]. With the increase in the number of cases of chronic Lyme disease, there is a growing urgency to discover how best to treat these patients. The limitations of “one variable at a time” sequential clinical trials include slow progress at a time when patients who are profoundly sick today do not have the luxury of waiting for tomorrow’s research. Fortunately, current technologies and large data sources provide us with the opportunity to develop newer, more robust, pragmatic trial designs to augment the rigor of randomized clinical trials and can accelerate the pace of research.

With the availability of larger databases, including patient registries such as MyLymeData, researchers have the opportunity to employ techniques to discover small to moderate treatment effects and then further examine the characteristics of the patients that are most responsive to treatment. This approach paves the way for analyzing patient characteristics to understand which patients are at higher risk of developing chronic disease and why a treatment is more effective with certain patients.

These factors, in turn, may be used to predict treatment responses in individual cases and to help address and prevent these differences from developing, for example, through early diagnosis and treatment. Possible approaches to teasing out treatment effects may include examination of the following: disease duration, disease severity at diagnosis, number of coinfections, patient demographics, and treatment delivery mechanism as well as treatment regimen and duration. A deeper understanding of treatment response may also help identify potential biomarkers for this disease and aid in developing more targeted and innovative treatments for those with chronic Lyme disease.

## Figures and Tables

**Table 1:** Demographic characteristics of respondents.

Variable	Count (% of Working Sample)
**Gender**	
Female	3250 (83%)
Mean age	49
**Education ^a^**	
High school or less	340 (9%)
Some college or associate degree	1265 (34%)
Bachelor degree	1139 (31%)
Graduate school degree	945 (25%)
**Family income ^b^**	
<$25k	485 (14%)
$25–50k	542 (15%)
$50–75k	547 (16%)
$75–100k	408 (12%)
>$100k	1025 (29%)
**Geography ^c^**	
East	1274 (33%)
Midwest	571 (15%)
South	1021 (26%)
West	1004 (26%)

^a^ 214 skipped or selected “prefer not to answer”. ^b^ 896 skipped or selected “prefer not to answer”. ^c^ Excludes 33 from a US territory or who did not indicate a state.

**Table 2:** Current stage of illness and diagnostic characteristics of sample.

Variable	Count (% of Working Sample)
**Current stage of illness**	
Chronic LD ^a^	61%
Late untreated LD ^b^	18%
Early Lyme disease ^c^	6%
Don’t know/Other	15%
**Stage when diagnosed**	
Late untreated LD ^b^	70%
Early Lyme disease ^c^	22%
Don’t know/Other	8%
**Key diagnostic factors**	
Clinician diagnosed (entry criteria for registry) ^d^	100%
Recollection of tick bite	41%
Recollection of EM rash ^e^	34%
With supportive lab tests	78%
1 or more coinfection	60%
Self-reported health status as fair or poor	65%
Disabled (with or without disability benefits)	32%

^a^ Remained ill for six months or more after treatment with antibiotics for 10–21 days. ^b^ Diagnosed and untreated for six months or more after symptom onset. ^c^ “Within days to weeks after my tick bite or exposure, I experienced symptoms associated with Lyme disease”. ^d^ To be enrolled, patients must have self-reported US residency and diagnosis by a healthcare provider. ^e^ Because of a branching error in the initial survey, patients were re-asked this question. This data includes the 1190 who responded to the revised question.

**Table 3:** Degree of change reported on global rating of change scale.

Better/Worse/Unchanged	Degree of Change	Likert Score	*n* (% of Total)	Assigned Group
**Better**	Hardly better at all	1	43 (1.22)	Low Responders
	A little better	2	269 (7.61)	Low Responders
	Somewhat better	3	298 (8.43)	Low Responders
	Total		17.26%	Low Responders
	Moderately better	4	295 (8.34)	High Responders
	A good deal better	5	450 (12.73)	High Responders
	A great deal better	6	289 (8.17)	High Responders
	A very great deal better	7	191 (5.40)	High Responders
	Total		34.64%	High Responders
	Total Better		51.9%	
**Unchanged ^a^**		0	1293 (36.57)	Nonresponders
**Worse**	A very great deal worse	−7	64 (1.81)	Nonresponders
	A great deal worse	−6	64 (1.81)	Nonresponders
	A good deal worse	−5	85 (2.40)	Nonresponders
	Moderately worse	−4	71 (2.01)	Nonresponders
	Somewhat worse	−3	66 (1.87)	Nonresponders
	A little worse	−2	35 (0.99)	Nonresponders
	Hardly worse at all	−1	23 (0.65)	Nonresponders
	Total Worse		11.54%	Nonresponders
**Total**			100%	

^a^ Includes “almost the same” (better/worse).

**Table 4:** Research trials for chronic Lyme disease are small and highly selective compared to patient-generated big data studies.

Study Type	Trial	Screened	Enrolled	Yield	Time to Recruit
RCT *	Klempner (2001)	1996	129	7%	3.3 years
RCT	Krupp (2003)	512	56	11%	2.5 years
RCT	Fallon (2008)	3368	37	1%	4 years
Big Data	Johnson (2014)	5357	3090	58%	6 months

* Randomized controlled trial [5,16,17,18].

## Supplementary Materials

The following are available online at http://www.mdpi.com/2227-9032/6/4/124/s1, Table S1: data used in the preparation of this article, obtained from the LymeDisease.org patient registry, MyLymeData, Phase 1 27 April 2017.

## Appendix A. MyLymeData Patient Registry Survey Overview

**Medical History**	**Patient Characteristics**
- Onset/duration - Severity - Diagnostic history/exposure - Treatment history - Medications - Diagnostic tests and results - Comorbidities - Reproductive history - Family history - Mortality (cause and date)	- Functional status, quality of life, symptoms - Marital status - Work status - Disability, work attendance (days lost from work), or absenteeism/presenteeism - Economic status - Healthcare resource utilization - Genetic information - Sources of care
**Treatment Risks and Benefits**	**Changes over Time**
- Medication, modes, duration of treatment - Adverse events - Treatment effectiveness	- Medications/medical status - Patient characteristics - Changes in financial status

## Appendix B. Potential Clinical Research Criteria for Pragmatic Big Data Trials

This appendix is derived from a paper by Stricker et al. [34] See Table 2 for diagnostic characteristics of sample.

- Required
  ○ Physician diagnosed
  ○ Clinical symptoms consistent with Lyme disease
  ○ Late stage Lyme disease (untreated for ≥6 months after symptom onset) or chronic Lyme disease (persistent symptoms for ≥6 months after treatment with <30 days of antibiotics)
- Strongly supportive
  ○ Positive serological testing
  ○ Fulfills CDC surveillance Western blot criteria
  ○ Fulfills Ma/Engstrom criteria
  ○ Seropositive for other tick-borne conditions
- Supportive
  ○ History of EM rash
  ○ Known or possible tick bite
  ○ Risk of exposure

## Appendix C. Data Sources and Limitations

**Data Source**	**Strengths**	**Weaknesses**
Claims Data	Ubiquitous Large Standardized	Insurance covered care only Designed for billing purposes No “results” Nonspecific Not timely
Academic case data	Well characterized samples Rigorous methodology Lab measures Assessments (Results)	EHR siloed Physician time constraints Practice constraints limit treatments Convenience sample
Community clinical data	Real-world clinical care N-of-1 practices Lab measures	EHR siloed or paper/nonexistent Physician time constraints Convenience sample
Patient registries	Real-world clinical care Standardized Timely Labor burden on patient Cross-silo	Emerging innovation Patient generated Self-selected sample Accuracy/Recall
